# Parameterisation of multi-scale continuum perfusion models from discrete vascular networks

**DOI:** 10.1007/s11517-012-1025-2

**Published:** 2013-01-24

**Authors:** Eoin R. Hyde, Christian Michler, Jack Lee, Andrew N. Cookson, Radek Chabiniok, David A. Nordsletten, Nicolas P. Smith

**Affiliations:** 1 Department of Computer Science, University of Oxford, Oxford, OX1 3QD UK; 2 Imaging Sciences and Biomedical Engineering Division, St Thomas’ Hospital, King’s College, London, SE1 7EH UK

**Keywords:** Parameterisation, Perfusion, Multi-compartment Darcy, Homogenisation, Discrete vascular anatomy

## Abstract

Experimental data and advanced imaging techniques are increasingly enabling the extraction of detailed vascular anatomy from biological tissues. Incorporation of anatomical data within perfusion models is non-trivial, due to heterogeneous vessel density and disparate radii scales. Furthermore, previous idealised networks have assumed a spatially repeating motif or periodic canonical cell, thereby allowing for a flow solution via homogenisation. However, such periodicity is not observed throughout anatomical networks. In this study, we apply various spatial averaging methods to discrete vascular geometries in order to parameterise a continuum model of perfusion. Specifically, a multi-compartment Darcy model was used to provide vascular scale separation for the fluid flow. Permeability tensor fields were derived from both synthetic and anatomically realistic networks using (1) porosity-scaled isotropic, (2) Huyghe and Van Campen, and (3) projected-PCA methods. The Darcy pressure fields were compared via a root-mean-square error metric to an averaged Poiseuille pressure solution over the same domain. The method of Huyghe and Van Campen performed better than the other two methods in all simulations, even for relatively coarse networks. Furthermore, inter-compartment volumetric flux fields, determined using the spatially averaged discrete flux per unit pressure difference, were shown to be accurate across a range of pressure boundary conditions. This work justifies the application of continuum flow models to characterise perfusion resulting from flow in an underlying vascular network.

## Introduction

Perfusion is essential for the healthy function of biological tissues. Blood flowing through the vasculature provides the means of metabolite exchange, delivery of energy sources, and removal of harmful by-products. The importance of the perfusion process is highlighted by the serious consequences of related pathologies, e.g. cardiac and cerebral ischaemia and infarction. A principal determinant of perfusion is the vascular anatomy, which is inherently multi-scale in nature. Previous computational simulations of blood flow have analysed the implications of this anatomy via statistically generated networks and rule-based networks [25, 33]. Such generated networks have been recently superceded by those derived from direct imaging techniques and subsequent image processing, e.g. micro-CT [12, 17] and cryomicrotome [37]. This latter technique has produced whole-organ vascular trees with vessels in the order of 10 μm in diameter.

While many 1D blood flow models have been successfully applied to large anatomical models [22, 26], there is a significant computational expense associated with the number of vessel segments in a whole-organ high resolution vascular model. Furthermore, the vascular anatomy obtained in a clinical setting typically reveals little beyond the largest epicardial vessels [14], and the determination of suitable intramural pressure/flow boundary conditions (BCs) is difficult. Hence, flow models that require detailed vascular and BC knowledge cannot be applied clinically due to this lack of available data. This paper outlines an approach to obtaining a flow solution that overcomes the issue of unknown BCs.

In this context, porous flow models, such as Darcy’s Law, provide a promising alternative framework, as evidenced by previous perfusion models for biological tissues [3, 30]. The Darcy model is simple in comparison to explicit nonlinear 1D flow models and it can account for the close spatial relation between vessels of disparate scales via the assumption of multiple spatially co-located porous domains [5, 16]. However, a suitable method for mapping multi-scale vessel data onto a set of point-wise parameters remains thus far unclear, with the exception of idealised situations, e.g. assuming a periodic material structure [4, 20]. A formal averaging theorem has been previously used to derive material parameters for porous flow models [9, 31]. However, this theorem is exact only in the limit of infinite vessels, and to date this approach has not been studied with regard to vessel density or the size of the spatial averaging volume. This volume, or representative volume element (RVE), is often used within spatial homogenisation techniques. Typically, the RVE is defined by the minimum volume within which the property of interest remains largely constant [2]. The technique of spatial averaging within an RVE is extensively applied within the Huyghe and Van Campen permeability parameterisation method. However, this method assumes a homogeneous distribution of the property of interest across the RVE, e.g. it is assumed that the vascular space is evenly distributed throughout the material. Experimental evidence and physiological knowledge suggest that this assumption is not valid at the scale of transmural and large arteriolar vessels [28]—hence the need for further analysis in this study.

Motivating the current work, we hypothesise that a significant proportion of 3D discrete vascular data can be incorporated into continuous porous flow domains via spatial parameterisation, and still provide a pressure solution that is sufficiently close to the discrete flow solution. For this application, we determine permeability tensor fields from the network morphology with the long-term goal of averaging these fields determined from several same-phenotype experimentally derived networks, in order to provide a clinically useful permeability field. However, the relation of the Darcy parameters, such as permeability, to the vascular anatomy is not clear. Furthermore, the level of anatomical detail required is also unknown. For these reasons, in this study, we directly address this issue of how to parameterise the Darcy domains using vascular anatomical data.

## Methods

### Model descriptions

#### Vascular model

**Fig. 1:**
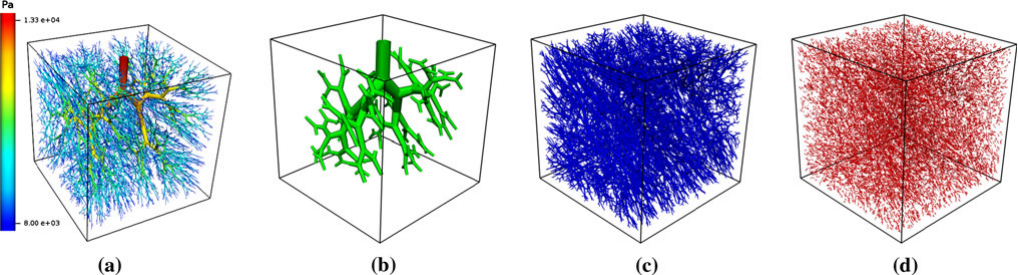
**a** Poiseuille pressure solution for the volume-filling synthetic network defined on $\Upomega = [-1, 1]^3\, \hbox{mm}$. The same vasculature is used to parameterise the Darcy model. **b**, **c** A partition of the vascular model in (**a**). Mean vessel radii of approximately 30, 6 and  3 μm were sought for compartments 1(**b**), 2(**c**), and 3(**d**), respectively

A mathematical representation of the vascular network is required to both parameterise and provide a comparison with the Darcy model outlined below. Typically the vascular model is derived from the image processing of experimental data [7, 37]. However, for the purposes of in silico testing in this study, a synthetic vascular model was created. In brief, the bifurcating, area-filling network generation of Wang and Bassingthwaighte [33] was extended to three dimensions [29]. Subdomain volumes were calculated using Monte Carlo integration [34]. Once the vessel centrelines distributed throughout the target domain $\Upomega=[-1, 1]^3\, \hbox{mm}$ were constructed, mass-conserving fluxes were assigned to vessels by assuming (1) all terminal vessels have a flow rate of 1, and (2) the flux through a parent vessel is equal to the total flux of its daughter vessels. Vessel radii were then assigned based on the following flux–radii relation

1 $$ r_i = [(q_i/\alpha_1)^{(1/\alpha_2)}] \alpha_3, $$

where *r*_*i*_ (*q*_*i*_) is the *i*th vessel radius (flux), and the following parameter values were used—α_1_ = 1, α_2_ = 3,  and  α_3_ = 0.001. Details of the lengths and radii of the created vessels can be seen in Table 1. This constructed model is used in preference to a more physiological model as it: (i) allows for a controlled definition of the ‘parent–daughter’ vessel radius relation and multi-scale aspects; (ii) is a fully connected network within a regular domain for ease of simulation. The vascular model consists of a set of vessels defined nodally, where each node encodes spatial and radial data and these fields are interpolated between the nodes by linear Lagrange basis functions (Fig. 1a).

**Table 1:** Compartmental statistics for the synthetic network used in the simulations of Sect. 3.1 and depicted in Fig. 1

Cpt #	No. of vessels	Length				Radius			
		Min	Max	Mean	SD	Min	Max	Mean	SD
1	221	0.0467	0.4999	0.1538	0.0608	0.0215	0.0961	0.0295	0.0120
2	32,645	0.0033	0.2547	0.0376	0.0217	0.0041	0.0216	0.0056	0.0022
3	32,867	0.0013	0.1608	0.0252	0.0150	0.0034	0.0037	0.0034	1.4e-5

We assume that the flow in each vessel is governed by Poiseuille’s law [15]. Specifically, the vessel flow rate, *Q*, is given by

2 $$ Q = \frac{\pi r^4}{8 \mu l}\triangle p, $$

where *r* is the vessel radius (assumed constant along the vessel, with value equal to the mean of the vessel nodal radii), *l* is the vessel length, μ is the dynamic viscosity of the fluid assumed throughout to be 0.0035 Pa s, and △*p* is the difference of the pressure between the vessel’s end nodes. Conservation of mass is enforced at each junction via

3 $$ \sum_{i \in T,j \in S} L_{ij} Q_j = 0, $$

where *T* is the set of internal nodes, *S* is the set of vessels, and the matrix **{L}** is defined to be

4 $$ L_{ij}= \left\{ \begin{array}{ll}+1, & \hbox {if for node }\,{\it i}, \hbox{segment}\,{\it j}\,\hbox{is incoming}.\\ -1, & \hbox {if for node}\,{\it i}, \hbox{segment} \,{\it j} \,\hbox{is outgoing}.\\ 0, & \hbox {if node} \,{\it i}\, \hbox{is not part of segment}\,{\it j}.\end{array} \right. $$

#### Darcy system

The multi-compartment Darcy system is a generalisation of the classical single compartment Darcy model to *N* porous compartments [5, 16]. The Darcy system for compartment $i \in [1,N]$ is^1^

5a $$ {\user2 {w}}_i + {\bf K}_i \cdot{\bf {\nabla} } p_i = 0 \,\hbox {in} \,\Upomega, $$

5b $$ \nabla \cdot{{\user2 {w}}_i} = s_i - \sum_{k=1}^N \beta_{i,k} (p_i-p_k) \,\hbox {in}\, \Upomega, $$

where subscripts *i* and *k* are compartment indices, ${\user2 {w}}$ and *p* denote the Darcy velocity and pore pressure, respectively, ${\user2{K}}$ is a permeability tensor of the material, *s* is a source field, and β is the array of inter-compartment coupling coefficients. The source field, *s*, can be spatially distributed to represent flow entering the Darcy compartment from proximal branches not included in the Darcy parameterisation. For the purposes of this study, however, it has been deemed to be an external factor to the Darcy parameterisation process and thus has not been included. An example of its intended use within a general perfusion modelling scenario can be seen in Michler et al. [16]. ${\beta_{i,k} \in \mathbb{R}_0^+}$ for $i,k=1,\ldots,N,$ and in order to conserve fluid mass across the system we require β to be symmetric. Thus, inter-compartment flow is provided for via volumetric source terms, scaled by the relevant pressure differences, whereas intra-compartment flow represents the flow for the set of vessels within a defined scale range and a given compartment. Further discussion on how the values of β are determined is deferred until Sect. 2.5.

For a general perfusion problem, Eq. (5) are set on an open bounded domain ${\Upomega \subset \mathbb{R}^{nd}}$ with spatial dimension *nd* and a piece-wise smooth boundary $\partial \Upomega.$ For the synthetic network simulations, $\Upomega = [-1, 1]^3\,\hbox{mm}$, and the solution is determined via the finite element method. The system is further complemented with zero flux BCs across $\partial \Upomega,$ thereby treating the tissue volume as an isolated block. Clearly this implies a compatibility condition for this static domain of

6 $$ \sum_{i=1}^N \int\limits_\Upomega s_i \, {\rm d}x = 0. $$

The multi-scale nature of vascular trees makes the use of a single porous compartment Darcy model inadequate if one is interested in the flow characteristics at the smaller scales due to the close spatial proximity of vessels with widely varying length and pressure scales. Thus, the approach taken is to employ multiple porous compartments with each compartment representing a different range of vascular scale, thereby reducing the variance of material properties per compartment.

Note that the simulations using the synthetic vasculature are not deemed to be general perfusion problems, and thus different BCs from those described above will be applied as described in Sect. 2.6.

### Vascular partition

In order to parameterise the Darcy domains, the vascular model is partitioned into associated distinct groups of vessels. In general, experimentally derived networks are not sufficiently regular to apply a simple metric such as radius for this partitioning purpose, as distal vessels in a non-idealised network may have a larger or similar radius in comparison to proximal vessels. Hence the need for a more robust partitioning metric that conserves the natural order of the discrete blood flow, i.e. proximal vessels should be used to parameterise proximal Darcy compartments. This metric, often called the *hierarchic parameter* ($\zeta$) field, is defined such that distal terminal nodes have $\zeta=0$ and the proximal terminal node has $\zeta=1.$ Thus, every point of the vascular model has $\zeta \in [0,1].$ In this work, the definition of $\zeta$ is based on the network morphology via a normalised distal vessel length metric, i.e. $\zeta$ at a node is the sum of the distal vessel lengths divided by the total network’s vessel length.

Hence, if one desires *N* porous compartments then *N* − 1 distinct, increasing $\zeta$ values are chosen, in addition to {0, 1}, to form an ordered vector *Z* of dimension *N* + 1. The *i*th vascular group is defined to be all vessels with average $\zeta$ value $\in [Z_i,Z_{i+1}).$ This same group is used to parameterise the permeability tensor field for the Darcy compartment *i*. Motivated by the network statistics (see Table 1), mean vessel radii of approximately 30, 6 and 3 μm were sought for compartments 1, 2, and 3, respectively. The use of a standard root-mean-square cost function based on the desired values yields a partition defined by the hierarchic parameter array of *Z* = [1, 0.019879, 0.000118, 0], whereby compartment *i* vessels are defined by those with average $\zeta$ value in the range [*Z*_*i*_, *Z*_*i*+1_) (Fig. 1b, c). Clearly the vascular grouping is likely to change if one uses a different metric to define the $\zeta$ field. However, if the parameterisation process is robust it will still be able to parameterise the Darcy fields for any other suitable vascular partition.

### Averaged discrete fields

An important assumption underpinning the hypothesis of this work is that the local vessels encode all the necessary information to determine the local permeability tensor. Therefore, in order to parameterise a point $\user2 {x} \in \Upomega$ for the Darcy compartment *i*, only the vessels of the associated vascular group and within the spherical RVE centred at $\user2 {x},$ denoted RVE($\user2 {x}$), are considered.

The same RVE is used to derive a spatially averaged discrete pressure for the *i*th Darcy compartment at $\user2 {x} \in \Upomega$ via

7 $$ \overline{p}_i(\user2 {x}) = \frac{\sum_{v \in V_i(\user2 {x})} P_v \,{\rm vol_v}}{\sum_{v \in V_i(\user2 {x})} {\rm vol_v}},$$

where $V_i(\user2 {x})$ is the set of vessels assigned to Darcy compartment *i* within RVE($\user2 {x}$), $vol_{v}$ is the volume of the vessel, and *P*_*v*_ is the average nodal pressure for the *v*th vessel within $V_i(\user2 {x}).$ The discrete pressure is averaged in this manner in order to provide a comparison with the continuum model pressures, as the discrete pressure is only defined along the vessel cross-sections whereas the Darcy model has a pressure defined over all of $\Upomega.$ The spatial averages of the other discrete fields of interest are defined in a similar fashion. The porosity of compartment *i*, denoted ϕ_*i*_ is defined to be

8 $$ \phi_i(\user2 {x}) = \frac{\sum_{v=1}^{V_i({\user2{x}})} {\rm vol_v} }{{\rm vol}_{{\rm RVE}(\user2 {x})}}, $$

where ${\rm vol}_{{\rm RVE}(\user2 {x})}$ is the volume of the RVE within $\Upomega,$ and thus the porosity of the material is

9 $$ \phi_f(\user2 {x}) = \sum_{i=1}^N \phi_i(\user2 {x}). $$

Finally, the value of the discrete mass flux from compartment *i* to *k*, where *i* > *k* without loss of generality, is also required. The set of all vessels within the RVE that belongs to compartment *k* but share a node with a compartment *i* vessel is denoted by *c*_*i*,*k*_. Then the flux traversing from compartments *i* to *k* is

10 $$ Q_{i,k}(\user2 {x}) = \sum_{v \in c_{i,k}(\user2 {x})} \frac{\pi r_v^4}{8 \mu l_v} (\bigtriangleup p_v). $$

### Determination of permeability

Without loss of generality, one assumes for the following discussion that there is a single porous domain with an associated distribution of vessels within it. The following three methods for parameterising the permeability tensor field are considered:

(i) *porosity-scaled isotropic* The vascular beds have frequently been treated as an isotropic porous medium [3, 8, 30], despite the fact that biological networks are rarely isotropic. Vessels with radii in the order of millimeters are clearly directional as their purpose is to rapidly distribute flow over a large volume [27], and capillaries with vascular radii in the order of microns are well-known to lie parallel to the myocytes in organised sheets [12]. Furthermore, the scale of the isotropic permeability is often taken to be constant across the domain, i.e.
  11 $$ \user2 {K}(\user2 {x}) = k {\user2 {I}}, $$
  where ${k \in \mathbb{R}^+, }$ and the identity tensor $\user2 {I}$ is assigned a unit of mm^2^ Pa^−1^ s^−1^. This approach fails to take into account known heterogeneities in vascular scale and density across the heart. In this work, we scale the identity tensor by the spatially varying porosity field, thus
  12 $$ \user2 {K}(\user2{x}) = \phi(\user2{x}) \user2 {I}, $$
  where ϕ is the porosity for this general domain. The benefit of including this method is twofold: (A) it is useful to quantify the error involved when making the isotropic assumption, and (B) it provides a reference solution against which the following two methods can be judged.
(ii) *Huyghe and Van Campen* Huyghe and van Campen (HvC) [9] derived an expression for a 4D (3D for intra-compartmental flow, and inter-compartmental flow constituting the extra dimension) permeability tensor based on the Slattery–Whitaker spatial averaging theorem [23, 36]. The formulation was for use in a deforming porous media model, and to the best of our knowledge, is the first attempt to utilise the detailed geometry of the underlying vascular network to parameterise the permeability tensor field. Notably, this formulation was only applied to a 2D idealised network. While the work of HvC focused on a 4D Darcy model and the derivation of the permeability field for this framework, we implemented the 3D spatial aspect of their 4D permeability tensor given entry-wise by:
  13 $$ K_{ij} = \frac{\pi}{128 {\rm vol}_{\rm RVE} \delta x_0 \mu} \sum_{ns} \frac{d^4 \bigtriangleup x_i \bigtriangleup x_j}{l}, \quad i,j=1,2,3, $$
  where δ*x*_0_ is an infinitesimal element of their hierarchic parameter, *ns* is the set of vessels within the hierarchic parameter range δ*x*_0_, and *d* is the vessel diameter. $\bigtriangleup x_i$ is the difference in spatial coordinate *i* between the vessel end points. Re-examining Eq. (2) and recasting the Poiseuille conductance, *C*_p_ in terms of vessel diameter
  14 $$ Q=C_{\rm p} \triangle P, \quad \hbox {where} \,C_{\rm p} = \frac{\pi d^4}{128 \mu l}, $$
  one can see that Eq. (13) makes the implicit assumption of Poiseuille flow in parallel vessels, with additional modifications for the creation of a permeability tensor. Crucially, the averaging theorem employed in their derivation is exact only in the limit of infinite vessel density, and until this work the results obtained for coarse networks using this method have not been shown to yield reasonable approximations vis-a-vis continuum and discrete pressure comparisons.
(iii) *projected-PCA*. Principal components analysis (PCA) is a technique of deducing orthogonal vectors that lie parallel to the vectors of largest data variance, and it is generally applied as a dimensional reduction tool for large data sets. For this study, the PCA technique has been specifically tailored for the purpose of permeability derivation from vascular networks. It provides a realistic alternative to the HvC method that can also yield anisotropic permeability fields. In order to determine $\user2 {K}(\user2{x}),$ the vessels within RVE($\user2{x}$) are first extracted. Each local vessel is translated such that the node closest to $\user2{x}$ is mapped to the origin, $\mathbf{0}.$ The vessel is re-scaled such that its length is equal to the value of the vessel’s Poiseuille conductance, *C*. In this fashion, a data set is constructed such that each vessel contributes one datum that is length *C* from $\mathbf{0},$ and this vector is oriented parallel to the original vessel direction. The zero-mean step of the PCA method is applied by duplicating each datum via reflection through $\mathbf{0}.$ The covariance matrix is then calculated from each dimension of the data set in the standard manner [10]. The resulting eigenvectors upon eigendecomposition of the covariance matrix are the principal components of the data set. Finally, the vessel orientation vector is projected onto the orthogonal eigenvector basis, the projections are normalised such that the sum of the projected lengths equal the vessel conductance, and then this normalised value is added to each associated eigenvalue of $\user2{K}(\user2{x}).$

We note that all of the above methods result in a symmetric positive definite tensor field, and have the correct physical units of mm^2^ Pa^−1^ s^−1^.

### Determination of pressure-coupling coefficients

With regard to the inter-compartmental conductivities, i.e. the β fields, one can continue with the Poiseuille flow analogy, as outlined in Eq. (10). If we consider the connectivity between two compartments, *i* and *k* say, then we can conclude that, per unit fluid density, we have

15 $$ \beta_{i,k} (p_i-p_k) = \tilde{q}, $$

where $\tilde{q}$ is a Darcy volumetric source, and so has units of mm^3^  *s* ^−1^ (see Eq.(5)b). This implies that β_*i*,*k*_ has units of mm^3^  Pa^−1^ s^−1^. It would be an open question as to the appropriate formulation for β if one utilised local vessel data only i.e. determine a form for $\Upgamma(r,l)$ which is defined to be the function of proportionality between β_*i*,*k*_ and the cross-sectional area of the vessels connecting the two Darcy compartments, or equivalently

16 $$ \beta_{i,k}(\user2{x}) = \sum_{v \in c_{i,k}} \pi r_v^2 \, \Upgamma(r_v,l_v). $$

**Fig. 2:**
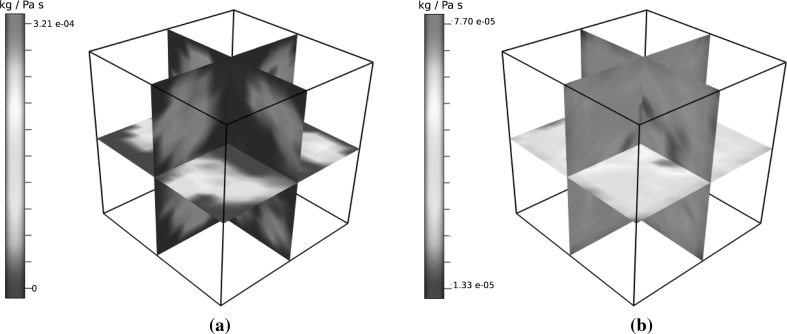
Using a three-compartment Darcy model requires three scalar pressure-coupling fields. Non-trivial values for any β_*i*,*j*_ such that |*i* − *j*| > 1 allow for coupling between non-neighbouring compartments *i* and *j*. By construction of the synthetic network, β_1,3_ is zero on $\Upomega.$ **a** β_1,2_ field. **b** β_2,3_ field

However, clearly the network pressure is a function of the *entire network morphology*. Thus, there may not exist such a function $\Upgamma,$ which depends on the local vessel morphology alone, that can adequately represent the inter-compartment coupling. Hence, we use the Poiseuille model to aid in the derivation of the β field, as the Poiseuille pressure field uses data from the entire network morphology in the construction of the Poiseuille network matrix [19]. From the perspective of the Darcy model, β_*i*,*k*_ can be viewed as the local constant of proportionality between the fluid flux transfer, and the difference of the Darcy pressures. Or, from the discrete model perspective,

17 $$ \beta_{i,k}({\user2{x}})=\left\{ \begin{array}{ll} 0 & \hbox {if}\,\overline{p}_i(\user2{x}) - \overline{p}_k(\user2{x})=0.\\ \frac{Q_{i,k}(\user2{x})}{|\overline{p}_i(\user2{x}) - \overline{p}_k(x)|}, & \hbox {otherwise}. \end{array} \right. $$

where *Q*_*i*,*k*_ is the spatially averaged mass flux between vessel groups associated to compartments *i* and *k* (Eq. (10)). Henceforth, the method of β determination used to produce the results presented in Sect. 3 is given by Eq. (17), and the fields themselves are visualised in Fig. 2.

### Simulation protocol

A root-mean-square (rms) error metric is employed to provide a method of comparison between the Darcy pressure, *p*, and averaged discrete pressure, $\overline{p}.$ The scalar pressure fields rather than the vector velocity fields are chosen for the simulation comparisons as it allows for the definition of a simpler and more transparent error metric. The effect of BCs used in the discrete/continuum models, in particular the difference between input and output pressures, on this metric should be reduced as much as possible. Thus, when comparing $\overline{p}$ to a spatially varying field *f* say, $\overline{p}$ is first normalised between 0 and 1. The same normalising transformation is applied to *f*, and the error between the two fields is defined to be

18 $$ {\rm rms}_{\overline{p}f} = \sqrt{\frac{\sum_{m=1}^c (\overline{p}(\user2{ x}^m) - f(\user2{x}^m))^2}{n}}, $$

where there are *n* points chosen from $\Upomega$ at which the two fields are compared, and $\user2{x}^m$ are the coordinates of the *m*th point to be compared. BC pressures of 13.3 and 8 kPa for the discrete model are applied at the root node and the remaining terminal nodes, respectively, for the 3-compartment simulations of Sect. 3. These values match the pressures applied by Vankan et al. [32], but it is worth stating here that the definition of the metric used to compare parameterisation methods normalises the pressure fields thus reducing the significance of the actual pressure BCs used (see Eq. (18)). In practice, the only fields compared to the averaged discrete pressure are the Darcy pressure (*p*), and the Poiseuille pressure ($\tilde{p}$) which is evaluated at the vascular model nodes. With respect to Darcy pressure comparisons, and unless otherwise stated, the set of points *n* is taken to be a regular grid distributed over $\Upomega$ with a grid spacing of 0.125 mm. For the Poiseuille pressure comparisons, *n* is defined to be a random selection of 1,000 vascular nodes, distributed over $\Upomega,$from the vascular compartment of interest.

The forthcoming simulations are focused on the issue of Darcy parameterisation, and hence they are not deemed to be general perfusion problems. Thus, BCs suited to this particular aim are applied. Specifically, zero flux BCs are imposed on compartment 2 as the Darcy velocity and pressure are being solved for in the usual manner. However, we do not solve for these quantities in compartments 1 and 3. Instead, Dirichlet pressure conditions are imposed across $\Upomega,$ and these are taken to be the averaged pressure values of the same vascular compartments via Eq. (7). In this manner, the testing framework compares the Darcy pressure for compartment 2 against the average discrete pressure of the vascular compartment 2 vessels, and uses the parameterised fields β_1,2_, β_2,3_ and $\user2{K}_2,$ thereby reducing our problem to the smallest number of parameterised fields. Note that $\beta_{1,3}({\user2{x}})=0 \; \forall \; {\user2{x}} \in \Upomega$ as the algorithm for constructing the synthetic network ensures that there is no connection between vessels of compartments 1 and 3.

Importantly, initial simulations revealed that none of the three permeability parameterisation methods provide the optimal scaling in terms of reducing the rms error. Thus in the following results sections, all $\user2{K}$ tensor fields are scaled by the constant that yields the lowest rms error for each individual method. The fact that our current solution system is simplified to just one compartment on which we solve for the Darcy state variables allows for the optimal scaling to be calculated via a gradient descent optimisation algorithm. The improvement in rms error afforded by this additional step is discussed further in Sect. 3.

## Results

### RVE radius

RVE size is an important parameter with respect to ${\rm rms}_{\overline{p}p},$ and a suitable size is dependent on the particular material application. For this application, it is dependent on the vascular scale and density of the Darcy compartment being parameterised, however, a suitable RVE radius is not obvious a priori. The RVE size should ensure a desired level of smoothness in the averaged field, but also capture the feature of interest.

**Fig. 3:**
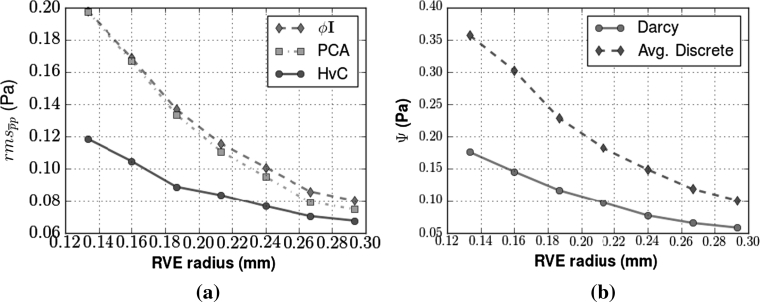
**a** Plot of the rms error between the discrete and continuum models for varying RVE radius comparing the three $\user2{K}$ parameterisation methods tested. **b** The reduction in the rms error is seen to be closely correlated with the smoothness of the Darcy (parameterisation via HvC method) and averaged Poiseuille pressure solutions. Our ‘unsmoothness’ metric, $\Uppsi,$ is defined via summation of the discrete Laplacian kernel, *A*, convolved with the local pressure array, $\overline{{\bf p}}_{ij},$ centred at the mesh node indexed by *ij* of the finite element model of $\Upomega,$ i.e. for the Darcy pressure we have ${\Uppsi=\sum_{i=1}^m \sum_{j=1}^m A*\overline{{\bf p}}_{ij}, \; \hbox { where }A,\overline{{\bf p}}_{ij} \in \mathbb{M}_{3 \times 3}}$ and there are *m* nodes in each dimension of the finite element mesh representation of $\Upomega$

**Fig. 4:**
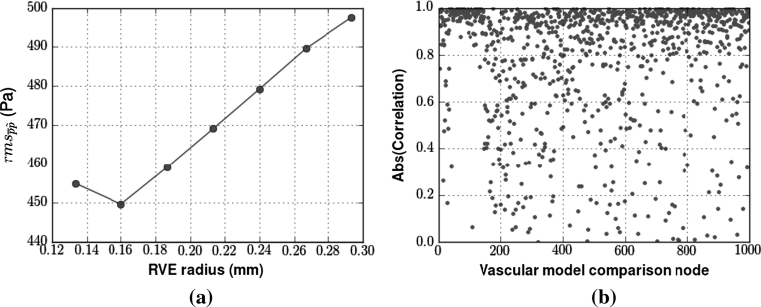
**a** Error measures demonstrating the difference between the actual nodal pressure of the discrete flow model, $\tilde{p},$ versus the spatially averaged pressure for a changing RVE radius. The error was calculated using a random selection of 1,000 vascular nodes from vascular compartment 2, and the $\overline{p}$ values evaluated at the same spatial coordinates. The linearity of the plot in **a** is due the high correlation of the individual nodal points with respect to the RVE size. The absolute value of correlation between the pressure from selected vascular nodal points and the RVE radius is shown in **b**

**Fig. 5:**
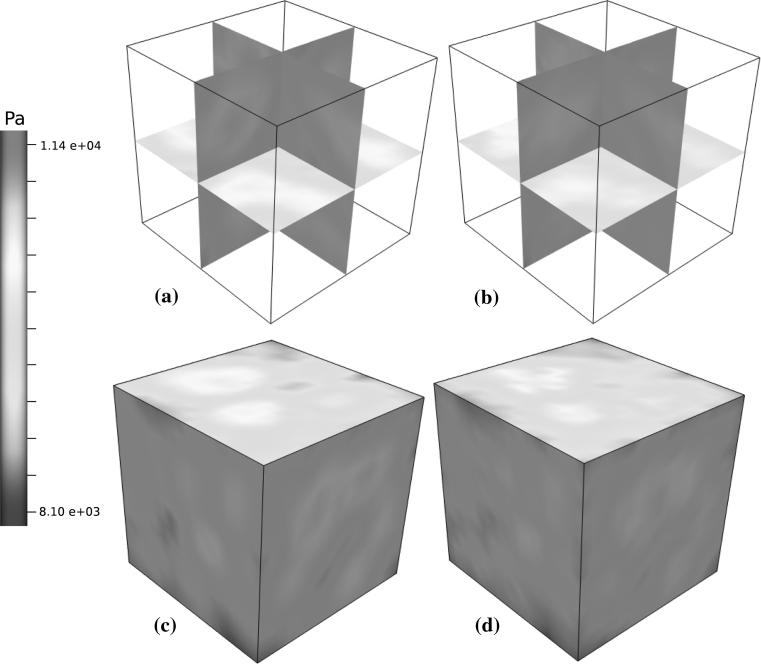
Darcy pressure results using the Huyghe and Van Campen method for determining $\user2{K}_2.$ **a** and **c** show the Darcy pressure field for internal isosurfaces and the external surface of $\Upomega,$ respectively. Similarly, **b** and **d** show the average discrete pressure field resulting from the Poiseuille model, also on the internal isosurfaces and the external surface of $\Upomega$, respectively

To investigate the effect of RVE radius on the continuum pressure solution, we evaluate ${\rm rms}_{\overline{p}p}$ for parameterisations defined using various RVE radii values. These results show that the error for all three parameterisation methods decreases with increasing radius (Fig. 3a). Moreover, the rate of decreasing error with increasing RVE radius is itself decreasing. This trend towards a plateau in error is to be expected, as both the spatially averaged pressure and permeability tensor value will tend towards the compartment average as the RVE radius increases. This convergence to the compartment average is demonstrated by the increased smoothness of the spatially averaged Poiseuille pressure (Fig. 3 b). The application of a larger averaging window allows the continuum solution, which is guaranteed to be smooth due to its use of interpolating functions, to better approximate the $\overline{p}$ field. We note with interest that the smoothness of the Darcy solution actually decreases from an RVE radius of 0.133–0.16 mm. Obviously, as the averaged pressure field becomes smoother with increasing RVE radius, the difference between the average discrete pressure field and the actual discrete pressure field existing on the vascular model increases. This is an important factor that is often overlooked during the application of spatial averaging techniques. Figure 4a demonstrates this general increase in error, and a clear linear trend is observed between ${\rm rms}_{\tilde{p}p}$ and the RVE radius. This trend is explained by the high correlation between the majority of the individual average discrete pressure values at the comparison points and the RVE radius used (Fig. 4b). The minority of nodes that do not display this high correlation must have one or more vessels in the periphery of the averaging window of significantly different pressure than that of the particular node itself. Yet again this trend does not hold within the RVE radius range of 0.133–0.16 mm. Thus, in choosing a RVE radius for a given vascular compartment, a suitable balance must be sought between increasing the smoothness of $\overline{p}$ and the divergence of $\overline{p}$ from the Poiseuille pressure $\tilde{p}.$ Furthermore, the RVE radius is bounded below by the smallest value that provides total volume coverage. In contrast to this result, there is no supremum bound on the size of the RVE, though of course one risks removing heterogeneities of interest at larger RVE sizes. In their paper, Vankan et al. [31] use a radius of approximately 13 % of the domain length. Of the radius lengths simulated, 0.2667 mm is the closest to this proportion and one can be confident that it is within a stable range of averaging window size. Figure 3 not only shows that the HvC method performs better than the other two methods with respect to the quantitative rms error metric, but also produces a qualitative match to the spatially averaged discrete pressure, as seen in Fig. 5.

### K parameterisation and vessel density

**Fig. 6:**
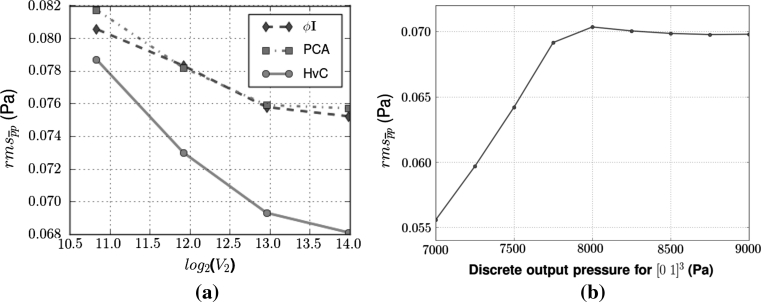
**a** Plot of the rms error between the discrete and continuum models for the three $\user2{K}$ parameterisation methods as the vessel density is varied. The axis displays the logarithm to the base 2 of the number of vessels in vascular compartment 2. **b** Terminal nodes in the subdomain *S* = [0, 1]^3^ mm were subjected to varying Dirichlet pressure BCs. The parameterisation of the Darcy model was done using a homogeneous output pressure of 8 kPa. Subsequently, only the Darcy Dirichlet pressure BCs are varied amongst the simulations. The decreasing rms error for output pressures <8 kPa is due to the normalisation step within the rms error estimation

The sensitivity of the $\user2{K}$ parameterisation methods with respect to vessel density is now examined via comparison of their ${\rm rms}_{\overline{p}p}$ values, as defined in Eq. (18). Not only should the optimum method have the lowest error, but it should also be robust against varying vascular partitions. Thus, Fig. 6a compares the errors over a varying vessel density range for the second Darcy compartment. The vascular partition specified by Fig. 1 yields a compartment 2 vessel radius range between 0.004 and 0.022 mm. The four data points for Fig. 6a were created by transferring vessels with radii less than 0.01, 0.008, 0.006 and 0.005, respectively, from compartment 2 into compartment 3.

There are three main points to note: (i) all three methods display decreasing rms error with increasing sampling domain radius, (ii) the HvC method consistently yields a significantly lower error than either of the other two methods, and (iii) the porosity-scaled isotropic method performed as well as the projected-PCA method. This latter point suggests that the synthetic network is inherently isotropic due to its volume-filling nature. We hypothesised that this last point occurred due to an intrinsic isotropic feature of the volume-filling network generation algorithm. In order to test this hypothesis, and apply our parameterisation process to a more realistic network, in Sect. 3.4 the three $\user2{K}$ parameterisation methods are applied to a confocal microscopy imaged rat capillary network [21].

### Independence from Poiseuille pressure boundary conditions

It remains to be shown that the HvC $\user2{K}$ and β parameterisation methods are robust against changes in the Poiseuille model BCs. The inter-compartment connectivity array β has been defined specifically with this need in mind, hence it uses local flux *per unit pressure difference*. As such, it should not require re-parameterisation when a change in pressure BC occurs, which would clearly be an untenable scenario for a dynamic large-scale perfusion model.

In order to demonstrate this independence, the pressure BCs at vascular terminal nodes in the subdomain *S* = [0,1]^3^ mm of $\Upomega$ are varied, while the remaining vascular terminal nodes are kept at 8kPa. Clearly $S \subset \Upomega = [-1,1]^3\,\hbox{mm}$ and moreover *S* represents a significant proportion of the tissue volume $\Upomega.$The Darcy parameter fields are derived with all vascular terminal nodes at 8 kPa. The pressure at the vascular terminal nodes within *S* is then varied over the range 7–9 kPa, with the only change in the Darcy model being the Dirichlet pressure BCs applied on Darcy compartments 1 and 3.

Two trends are visible in Fig. 6b. Firstly, and most importantly, there is no increase in the rms error across the boundary pressure range tested, in particular for 8–9 kPa. This demonstrates the independence of the parameterisation from the discrete BCs used to define the β array. Secondly, the decrease in rms error for boundary pressures less than 8 kPa is explained by the normalisation step in calculating the rms error. As the averaged discrete pressures are spread over a relatively wider range for these boundary pressure values, the normalisation step decreases the variance of the pressure discrepancies, thereby yielding a rms error that decreases in conjunction with the linearly decreasing discrete pressure BC.

### Anisotropic networks

**Fig. 7:**
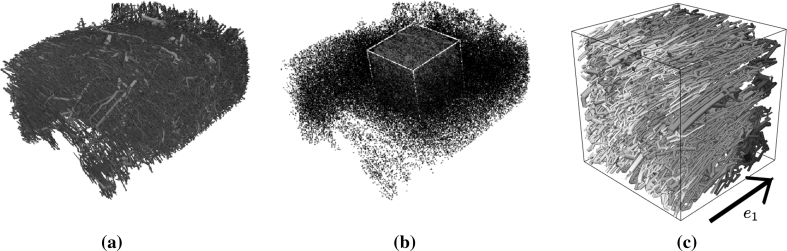
**a** A vascular model resulting from a confocal study of a rat LV tissue wedge. **b** The depicted cube shows the location of the extracted vessels of Case 1 in relation to the sample in **a** (see also Table 2). **c** Poiseuille pressure was solved on the extracted subnetwork with pressure BCs on two opposing faces (input pressure of 2 kPa and outflow pressure of 1 kPa), and no flux conditions on all other terminal nodes. Note that the subnetwork has been rotated from the view in **b**

To further examine the merits of each $\user2{K}$ parameterisation method, we consider the anisotropic network shown in Fig. 7. This vascular model was constructed from confocal data of a rat left ventricle tissue wedge, with average radii of 5.3 ± 2.3  μm [13]. This network has a clear preference of vessel direction, with the vessels (predominantly capillaries) being generally aligned with the local myofibres, as well as being organised into inter-weaving sheets. A cube of side length 0.67 mm (dictated by the overall block size) was selected from the block, and all its connected components were extracted to form a subnetwork. The extracted subnetwork is not partitioned into multiple vascular groups as (i) the vast majority of vessels are of the same scale, and (ii) testing the hypothesis that the introduced anisotropy will separate the projected-PCA and porosity-scaled permeability parameterisation methods, with respect to ${\rm rms}_{\overline{p}p},$ can be done without a multi-compartment system. Pressure BCs of 2 and 1 kPa were assigned to all terminal nodes on two opposing faces, the remaining terminal nodes are constrained to zero flux. These pressure values are motivated by being close to typical capillary pressures, but as stated previously, the actual values used are not significant due to the normalisation step in the definition of the rms value (see Eq. (18)). The averaged Poiseuille pressure was calculated at regular grid points, which was simultaneously parameterised using the various $\user2{K}$ parameterisation methods. In order to match these conditions as closely as possible in the Darcy model, the face *e*_1_ = 0 has a Dirichlet pressure BC value of 2 kPa and the opposing face has a Dirichlet pressure BC value of 1 kPa (see Fig. 7c). The remaining four faces have zero flux conditions imposed. These BCs reflect the fact that the Poiseuille flow is constricted by virtue of its own BCs to yield a predominant flow parallel to the local *e*_1_ axis, and flow can only leave the domain through the face *e*_1_ = 1.

The results displayed in Table 2 demonstrate that once more the HvC method has the lowest ${\rm rms}_{\overline{p}p}$ error of the three methods. Furthermore, a significantly smaller rms error is obtained from the projected-PCA method in comparison to the isotropic assumption, suggesting that the local vessel directionality is more effectively captured by the former method.

**Table 2:** $rms_{\overline{p}p}$ values for the anisotropic networks extracted from confocal imaging data of a rat left ventricle tissue wedge

Case	$\phi\mathbf{I}$	PCA	HvC
1	1.239e-01	1.085e-01	9.330e-02
2	1.189e-01	1.049e-01	9.773e-02

Observe that the projected-PCA method has outperformed the porosity-scaled isotropic permeability assumption. Case 1 is illustrated in Fig. 7. Case 2 is another extracted subnetwork from the wedge that does not share any vessel with the Case 1 network

## Discussion

In this study, 3D Darcy porous domains have been successfully parameterised using 1D vascular data and a combination of new and previously existing averaging methods. Both idealised and physiologically realistic networks were used to quantitatively compare the three methods to the average Poiseuille pressure via a root-mean-square error metric. The quantitative and qualitative results of Figs. 3 and 5, respectively, justify the original hypothesis, i.e. that a Darcy pressure within a reasonable error tolerance with respect to a discrete pressure model can be obtained via spatial averaging of 3D vascular networks. In practice, a ‘reasonable’ value of the error tolerance is application dependent and when modelling perfusion in a more realistic scenario, e.g. using a whole-organ anatomical vascular model, it may be more relevant to consider the error propagated through to the Darcy velocity fields. We note that PCA has been successfully used for determining material parameters within geomechanics studies [6, 11]. A key strength of PCA is its ability to easily and robustly extract the primary directions along which the data varies the most. In relation to flow modelling, these directions are naturally the principal flow directions. Despite this clear connection to permeability tensors, the HvC method was shown to be the optimum method across the range of our simulation study, which included varying vessel density and RVE size. This is most likely due to its rigorous mathematical foundation, but importantly, it was also better for relatively coarse vascular networks where the assumptions underlying the HvC method may not hold (see Sect. 2.4(ii)), particularly the homogeneous distribution of the averaged fields within the RVE. Clearly the vascular densities simulated were within a range where the HvC method is applicable.

Furthermore, we have characterised a significant proportion of the rms error to vessel density relationship for our synthetic network. The vascular density results of Fig. 6a indicate that this property of the underlying network is a key factor in deciding a priori what parameterisation method to apply, or indeed whether or not the continuum approach is appropriate in the first instance. Varying vascular density as per Sect. 3.2 is effectively equivalent to altering the hierarchic parameter vector entry *z*_3_. This demonstrates the robustness of the parameterisation method for this particular network, and a more comprehensive study of hierarchic parameter vector perturbation is currently underway on an anatomical vascular model. We also showed that the derivation of the inter-compartment coupling fields β was robust in relation to discrete pressure BCs. This is a critical finding with respect to the future application of the Darcy parameterisation to dynamic perfusion models.

The challenging issue of choosing the optimal number of Darcy compartments, *N*, is very much problem dependent. With respect to whole-organ anatomical vascular models, the choice of *N* is limited by the number of vessels obtainable from the experimental data and image processing. In this sense it is related to the vascular density, the applicability of Darcy’s Law, and the impact of *N* on the overall perfusion results.

We believe that our 3D Darcy model plus volumetric sources for inter-compartment connectivity is more representative of the perfusion process in tissues than the pioneering 4D Darcy model of Huyghe and Van Campen [9] as we use global network data in the definition of the inter-compartment coupling field β (see Eq. (17)). While they do use a discrete pressure field in the definition of one of their hierarchic parameter fields, this merely determines which vessels belong to each compartment, and is not used in the determination of the parameter values themselves.

### Limitations

The application of Darcy’s Law to a branching network does not strictly satisfy the assumptions of its derivation via homogenisation, i.e. the requirement of a spatially periodic microstructure. However, one should recall that Darcy’s Law originated as a phenomenologically derived constitutive equation. The merits of applying a Darcy approach for perfusion modelling should be judged by the simulation outcomes. Of course, one could assume that as the vessel scale increases one is moving further away from a state where it is appropriate to apply the Darcy model. At which point this transition occurs is currently being investigated.

In common with many spatial averaging techniques, a limitation of our parameterisation process is the choice of RVE size. In Sect. 3, Fig. 3 shows a decrease in rms error with increasing smoothness of the averaged discrete pressure. Concomitantly, Fig. 4 reveals the divergence of the averaged discrete pressure from the true Poiseuille solution. The decreased smoothness of the Darcy solution from RVE radius of 0.133–0.16 mm is an indication of fluctuations in the parameterised fields. This is an undesired feature within the spatial averaging technique, and thus an RVE of this size is deemed to be unsuitable. We suggest that the RVE size should be chosen based on analysis of these two relationships to yield an RVE based upon a desired error tolerance and faithfulness to the discrete pressure over the network. The RVE should also be of a sensible size with respect to any available clinical validational data, e.g. the RVE radius should be smaller than the in-plane resolution of the perfusion imaging data (typically with an in-plane resolution of 1–1.5 mm), as otherwise the model is averaged over a larger volume than the data we wish to compare to. Moreover, an unsuitably large RVE size can produce various undesired artifacts, e.g. constant porosity across $\Upomega,$or the creation of unphysiological flow paths. The error metric results presented have been computed on a 16 × 16 × 16 mesh. To test for convergence, the Darcy parameterisation methods were applied to a finer mesh (32 × 32 × 32), the results of which were found to be qualitatively the same as those on the coarser mesh (results not shown).

RVE size is also sensitive to the hierarchic parameter, $\zeta,$ and hence the vascular partition. The $\zeta$ field relies on the quality and type of the vascular model. A poorly segmented network with missing subtrees would yield vessels with unusually small $\zeta$ value compared to vessel radius. Of course these could be easily detected via statistical tests on the network. If vascular loops are present in the arterial network model then $\zeta$ could be defined via a discrete pressure solution [32].

As stated previously in Sect. 3, all $\user2{K}$ tensor fields have been individually scaled by a constant in order to yield the lowest rms error for each method. While this is not a serious computational burden, work is ongoing to develop an analytically derived scale that is dependent on the vascular anatomy with the aim of making this post-processing step redundant or at least obtaining a closer starting point for the optimisation algorithm. The fact that a reduction in rms error is achieved by this step is indicative that the validity of the assumptions implicit in the $\user2{K}$ parameterisation methods may not strictly apply. One could consider the rescaling as a manner of accounting for the facts that there are a finite number of vessels in the compartment and that not all vessels are actually in parallel. Furthermore, rather than being a hindrance, the exciting possibility of using the $\user2{K}$ rescaling in the future as a metric to guide the optimisation of the number of compartments to be employed is being investigated.

### Towards clinical relevance

The perfusion model presented currently considers flow within the closed section of the circulatory system only, i.e. all fluid mass that enters the tissue exits via the venous system. The application of our perfusion model is focused on flow behaviour over the relatively short time frame of 1 or 2 s, and thus the effect of the lymphatic system is neglected. However, we recognise the importance of the lymph system for understanding a number of known pathophysiologies. Thus, if perfusion is, in future, modelled over a longer time frame, such as a day, then extra porous compartments could be added to model lymphatic flow, provided suitable experimental data can be obtained to parameterise the permeabilities of these additional compartments.

We verified that the porosity-scaled isotropic method produced larger rms errors, in comparison to methods that used directional vessel data, when anisotropy was known to be present in the network. However, it is worth noting that this error converges towards the HvC error with increasing RVE size (Fig. 3), yielding a 22 % increase in the porosity-scaled isotropic error over the HvC error at our preferred RVE radius of 0.2667 mm. This could allow for the use of the less complex porosity-scaled isotropic method for parameterising models where detailed vascular data is not available. In the near future, it may even be possible to determine in vivo total macroscale porosity measures of tissue, using a combination of high resolution MRI and intravascular contrast agents.

This paper outlines a method for parameterising a static perfusion model. Clearly $\user2{K}$ would need to alter if the network undergoes deformation and/or dynamic changes in intramyocardial stress, as one would expect in a beating heart [24]. Geometric changes can be accounted for within this process by appropriate transformations utilising the deformation gradient tensor and porosity changes. Providing for realistic coupling between the mechanical stress and intravascular pressures is likely to involve complex extensions to the usual strain-energy constitutive equations. Modelling autoregulation, within a dynamic perfusion model, represents a more formidable challenge. Such an addition would increase the complexity and non-linearity of the model significantly, but incorporation of this sympathetic response is a long-term goal for the future modelling of tissue perfusion.

While this paper focuses on a tissue domain that is perfused by a single network, our methods naturally extend to the case of multiple distinct perfusion regions existing within a contiguous tissue domain. Evidence for such distinct perfusion regions has been provided by direct visualisation of cryomicrotome data [28]. Indeed, in a previous paper, a single arterial subnetwork was extracted for use in a perfusion model from a whole-organ arterial tree [5]. The obvious extension to this work is to simulate perfusion within a tissue volume supplied by numerous arterial subnetworks whereby the Darcy domains are spatially partitioned into the distinct perfusion regions. This model can easily be extended to consider a subdomain of the organ volume, and in this scenario the zero flux BCs could be relaxed, e.g. to simulate the presence of a collateral vessel.

In recognition of its critical role in organ perfusion and pathological perfusion diseases, the microcirculation networks can be incorporated into the perfusion model as another Darcy compartment. However, while there is a lack of whole-organ anatomical capillary network data, this could be overcome via estimating vessel directions through correlations with tissue structural data such as the local fibre/sheet/sheet-normal axes. Principal flow directions within the microcirculatory compartment, defined using this existing data, would be an important addition to our perfusion model.

Once fundamental parameterisation questions have been resolved, one can then simulate more clinically realistic scenarios, using patient-specific, CT-derived large epicardial vessel models to provide the volumetric source driving the multi-compartment Darcy perfusion model. The latter component of which has already been shown to be able to be simulated within a clinically relevant timescale [16].

Finally, throughout this study, the tissue and accompanying vascular network have been treated in a general manner. Thus, this method and the findings with respect to sampling domain size and vessel density sensitivities are applicable to perfusion models of various tissue types which are clearly of interest, e.g. lung [18], kidney [35], brain [1], etc. Intra-species variability of same organ and same phenotype vascular networks has yet to be studied on a statistically significant scale, primarily due to the expense of such a survey. Nevertheless, this is an important future step if we wish to utilise averaged permeability fields within a generic perfusion model of the given organ. Ultimately, the combination of this multi-compartment Darcy model, an experimentally derived vascular model, and extracted microsphere data from the same tissue volume used to provide the vascular model would potentially form the ideal experimental validation of our methodology.

## Footnotes

1 Einstein summation is not in use
